# Genetic Modulation of c-di-GMP Turnover Affects Multiple Virulence Traits and Bacterial Virulence in Rice Pathogen *Dickeya zeae*

**DOI:** 10.1371/journal.pone.0165979

**Published:** 2016-11-17

**Authors:** Yufan Chen, Mingfa Lv, Lisheng Liao, Yanfang Gu, Zhibin Liang, Zurong Shi, Shiyin Liu, Jianuan Zhou, Lianhui Zhang

**Affiliations:** 1 Guangdong Province Key Laboratory of Microbial Signals and Disease Control, South China Agricultural University, Guangzhou, People’s Republic of China; 2 Integrative Microbiology Research Centre, South China Agricultural University, Guangzhou, People’s Republic of China; 3 State Key Laboratory for Conservation and Utilization of Subtropical Agro-Bioresources, Guangzhou, 510642, China; Shanghai Jiao Tong University, CHINA

## Abstract

The frequent outbreaks of rice foot rot disease caused by *Dickeya zeae* have become a significant concern in rice planting regions and countries, but the regulatory mechanisms that govern the virulence of this important pathogen remain vague. Given that the second messenger cyclic di-GMP (c-di-GMP) is associated with modulation of various virulence-related traits in various microorganisms, here we set to investigate the role of the genes encoding c-di-GMP metabolism in the regulation of the bacterial physiology and virulence by construction all in-frame deletion mutants targeting the annotated c-di-GMP turnover genes in *D*. *zeae* strain EC1. Phenotype analyses identified individual mutants showing altered production of exoenzymes and phytotoxins, biofilm formation and bacterial motilities. The results provide useful clues and a valuable toolkit for further characterization and dissection of the regulatory complex that modulates the pathogenesis and persistence of this important bacterial pathogen.

## Introduction

*Dickeya zeae* is the causal agent of bacterial foot-rot disease in rice planting countries and regions including China and other southeast Asian countries. As a novel genus reclassified from *Erwinia chrysanthemi* in 2005[1], most species in *Dickeya* genus infect dicotyledons, however, *D*. *zeae* strain EC1 is one of a few strains that can infect both monocotyledons and dicotyledons[2–4], suggesting that *D*. *zeae* EC1 may have evolved new pathogenic mechanisms to broaden its host range. Within the *Dickeya* genus, *D*. *dadantii*, represented by strain 3937 (*E*. *chrysanthemi* 3937), is one of the most characterized species[5], which can cause soft rot disease in numerous cash crops. It produces various virulence factors including exoenzymes, mainly pectate lyases (Pel), proteases (Prt), celluloses (Cel) and their isoenzymes[6–8], as well as Type III secretion system (T3SS)[9, 10]. In contrast, the major virulence determinants of *D*. *zeae* strain EC1 appear to be a family of phytotoxins collectively known as zeamines[11], as mutation of the genes encoding zeamine production drastically reduced the bacterial virulence on rice and dicotyledon plants[12, 13]. In addition, the null mutant of AHL quorum sensing signal synthase showed increased bacterial motility and decreased biofilm formation, and marginally attenuated virulence on host plants[4]. Although some virulence factors are characterized, the molecular mechanisms and signaling pathways which govern the virulence gene expression in *D*. *zeae* remain largely undetermined.

C-di-GMP is a universal second messenger regulating a range of important cellular processes in many bacterial pathogens, including, but not limited to biofilm formation, flagella-mediated motility, and production of numerous virulence factors. Since its first discovery nearly 3 decades ago[14], substantial progress has been made in understanding its signaling networks and regulatory mechanisms[15]. The turnover of c-di-GMP molecules in bacterial cell is contingent on two types of enzymes with opposite functions i.e., diguanylate cyclase (DGC) and phosphodiesterase (PDE). DGCs with a GGDEF motif are used to synthesize c-di-GMP, while PDEs degrade c-di-GMP into 2 GMPs or pGpG according to the canonical motif HD-GYP or EAL, respectively [16–18]. It is well recognized that the homeostatic status of intracellular c-di-GMP could influence numerous bacterial physiological and biological functions, including cell morphology, expression of virulence genes, and cell differentiation[15]. In some model species, such as *Pseudomonas aeruginosa* and *Xanthomonas campestris* pv. *campestris* (*Xcc*), the genes encoding c-di-GMP metabolism have been intensively investigated[19–24]. However, even in these well-characterized bacterial species, the complexity of c-di-GMP signaling networks is still far from clear, leaving many questions to be answered.

In *D*. *dadantii* strain 3937, two PDE proteins EcpB and EcpC were reported to regulate genes encoding extracellular enzymes and type III secretion system (T3SS), and act as a global regulator modulating bacterial motility and biofilm formation[25]. To understand the signaling regulatory mechanisms that control the virulence of *D*. *zeae*, we recently completed the genome sequencing of *D*. *zeae* strain EC1[26]. In this study, we constructed in-frame deletion mutants targeting all the predicted c-di-GMP related genes by using strain EC1 as a parental strain. Phenotypic assessments, including biofilm formation, swimming and swarming motility, production of cell wall degrading enzymes and phytotoxin zeamines, were conducted on the mutants. The results provide useful information on the roles of c-di-GMP metabolic genes in the modulation of the bacterial physiology and virulence, and present clues and resources for further investigation of the c-di-GMP signaling mechanisms in this important bacterial pathogen.

## Materials and Methods

### Bacterial strains and plasmids

Bacterial strains and plasmids used in this study are listed in Supplemental Material (S1 and S2 Tables). *Escherichia coli* was routinely grown at 37°C in Luria—Bertani (LB) medium (each liter contains 10 g Bacto tryptone, 5 g yeast extract, and 10 g NaCl, pH7.0). *D*. *zeae* EC1 and its derivatives were grown at 28°C in LB medium as indicated[4]. For testing the antibiotic production, bacteria were grown in the optimized medium LS5 with minor modifications (each liter contains 5.25 g K_2_HPO_4_, 2.25 g KH_2_PO_4_, 10.0 g sucrose, 3.6 g NH_4_NO_3_, 1.0 g KCl and 0.25 g MgSO_4_, pH 7.0)[27]. The cell aggregation inducing medium, SOBG was modified from SOB (each liter contains 20 g of tryptone, 5 g of yeast extract, 2.4 g of MgSO_4_, 0.5 g of NaCl, 0.186 g of KCl) plus 2% glycerol [10]. Antibiotics were added at the following final concentrations when required: ampicillin (Ap), 100 μg/ml; kanamycin (Km), 100 μg/ml; streptomycin (Str), 25 μg/ml; and polymyxin (Pm), 40 μg/ml. The optical density (OD_600_) of the bacterial culture was measured by using a UNIC 7200 spectrophotometer at 600 nm.

### Mutant construction and complementation

In-frame deletion of the genes encoding GGDEF and EAL domain proteins was generated by allelic exchange[28]. The flanking region of each gene was amplified by PCR using the specific primers listed in Supplemental Material (S3 Table). The PCR product was then digested with *Bam*HI-HF and *Spe*I-HF (New England BioLabs, MA), and cloned into suicide plasmid pKNG101 digested with same restriction enzymes. The resulting constructs were transformed into *E*. *coli* CC118 λ-*pir*, individually, and then mobilized into *D*. *zeae* EC1 by triparental mating using the helper strain HB101 (RK2013). Recombinants were selected on the medium containing sucrose (5%), and mutations were confirmed by PCR and DNA sequencing.

For complementation analysis, the coding region of the target gene was amplified by PCR using specific primers (S3 Table). The PCR fragments were cloned into the plasmid pBBR1MCS-4 at *Hind*III-HF/*Bam*HI-HF or *Eco*RI-HF/*Bam*HI-HF sites. The resultant construct was transformed into *E*. *coli* DH5α and mobilized into mutants by conjugal triparental mating. The complemented strains were confirmed by PCR analysis and DNA sequencing.

For overexpression of the genes encoding the GGDEF and EAL domain (S2 Fig), the *wspR* and *rocR* genes known to encode functional enzymes[29, 30], were amplified from *P*. *aeruginosa* PAO1, respectively. These PCR fragments were cloned into the broad-host-range vector pBBR1-MCS4 as described in the previous section. The resultant overexpression constructs were respectively introduced into *D*. *zeae* EC1 competent cells by heat-shock and transformants were selected in LB plate containing ampicillin (100 μg/ml).

### Motility assay

Swimming motility was assessed in the semisolid medium plate, which contains about 15 ml of semisolid Bacto tryptone agar medium (each liter contains 10 g Bacto tryptone, 5 g NaCl, and 3 g agar) supplemented with 0.05% (w/v) 2,3,5-triphenyltetrazolium chloride (TTC) for visualizing the colony[31]. The bacterium was either spotted using a toothpick or inoculated with 1 μl of an overnight bacterial culture. Plates were incubated at 28°C for 28 hours before measurement. Swarming plate was prepared by pouring 15 ml of specific medium (Tryptone 10g; NaCl 5g; agar 4g per liter) and left to dry at room temperature for about 30 min without covering in a biosafety cabinet, then kept at room temperature overnight. The bacterium was inoculated in the middle of the plate using a toothpick. Plates were incubated at 28°C for 24 hours, and the diameter of the bacterial radial growth was measured[32].

### Biofilm formation and quantification

Overnight bacterial culture was diluted in 1:100 in SOBG or LB medium. For quantification of biofilm formation, 100 μl of diluted culture was transferred to each well of 96-well polypropylene microliter plates. The plates were incubated at 28°C with shaking at 150 rpm for 18 h. Cultures were then removed and added with 200 μl of 0.1% crystal violet (w/v). After stained at room temperature for 15 min, the dye was removed and the wells were rinsed for three times with water. For quantification of the attached bacterial cells, the stained wells were decolorized with 200 μl of 95% ethanol after drying. The quantity of crystal violet was determined by measuring the absorbance at 570 nm.

### Extracellular enzyme activity assays

Bacterial growth conditions, preparation of culture supernatants and assay conditions for Pel, Prt, Cel and polygalacturonase (Peh) were described previously with minor modifications[33]. Media for enzyme activity assays were as follows: Pel medium [each liter contains 10 g polygalacturonic acid (Sigma), 10 g yeast extract, 0.1125 g CaCl_2_, 100 mM Tris-HCl and 8 g agarose, pH 8.5], Prt medium [LB medium containing equal volume of 1% (v/v) skimmed milk], Cel medium (each liter contains 1.0 g carboxymethyl cellulose, 3.8 g Na_3_PO_4_ and 8.0 g agarose, pH 7.0), Peh medium [each liter contains 5.0 g polygalacturonic acid (Sigma), 2.0 g sucrose, 15.0 g agar and 2.0 g (NH_4_)_2_SO_4_, PH 5.5)]. For semiquantitative assays, wells were punched in each plate with a 5 mm cork borer. Twenty microliters of bacterial culture (OD_600_ = 1.5) were added to the wells and incubated at 28°C. Pel and Peh assay plates were developed with 1 N HCl after 11 h and 13 h, respectively. Detection of cellulase activity was performed by Congo red (0.1%) staining and 1 M NaCl decoloration. Halos around the wells became visible in Prt assay plates in 20 h without any further treatment. All assays were repeated for three times and the data were presented as mean ± standard deviation (SD).

### Zeamine production assay

Zeamine production assay was carried out in LS5 medium. Plates were prepared by pouring about 20 ml of LB agar medium, overlaid with 5 ml of 1% agarose containing about 1.0 × 10^8^ cells of fresh *E*. *coli* DH5α after solidification. The filter-sterilized supernatants of bacterial cultures (OD_600_ = 1.5) in LS5 medium (20 μl from each sample) were added to the wells in plates. The bioassay plates were incubated at 28°C for 24 h before photography. The radius of the inhibition zone was measured and the toxin units were transformed according to the toxin formula: zeamines (units) = 0.5484^e0.886x^ (R^2^ = 0.9957), X is the width in millimeters of the growth inhibition zone surrounding each well[27].

### Bacterial growth analysis

Bacterial growth assay was performed by using 96-well polypropylene microliter plates. Overnight bacterial cultures grown in LB broth were diluted in 1:100 with three types of media used in this study. One hundred microliters of inoculated culture were transferred into each well at 28°C with 150 rpm shaking. The optical density at 600 nm of bacterial culture in LB and LS medium was measured at different time points (4, 8, 12, 22, 26 and 30 h) using Synergy H1 Multi-Mode Reader (BioTek). The same assay was performed in SOBG medium and the optical density of bacterial cells was measured at 8-time points (4, 8, 12, 14, 18, 22, 26 and 30 h).

### Statistic analysis

Experiments were repeated in triplicates. *, P < 0.05 (Student’s *t*-test). The paired two-tailed Student’s *t*-test was performed between the wild type EC1 and each derivative strain, by using GraphPad Prism 5.0 software (GraphPad, La Jolla, CA).

## Results

### Construction of a collection of c-di-GMP turnover gene mutants

**Fig 1:**
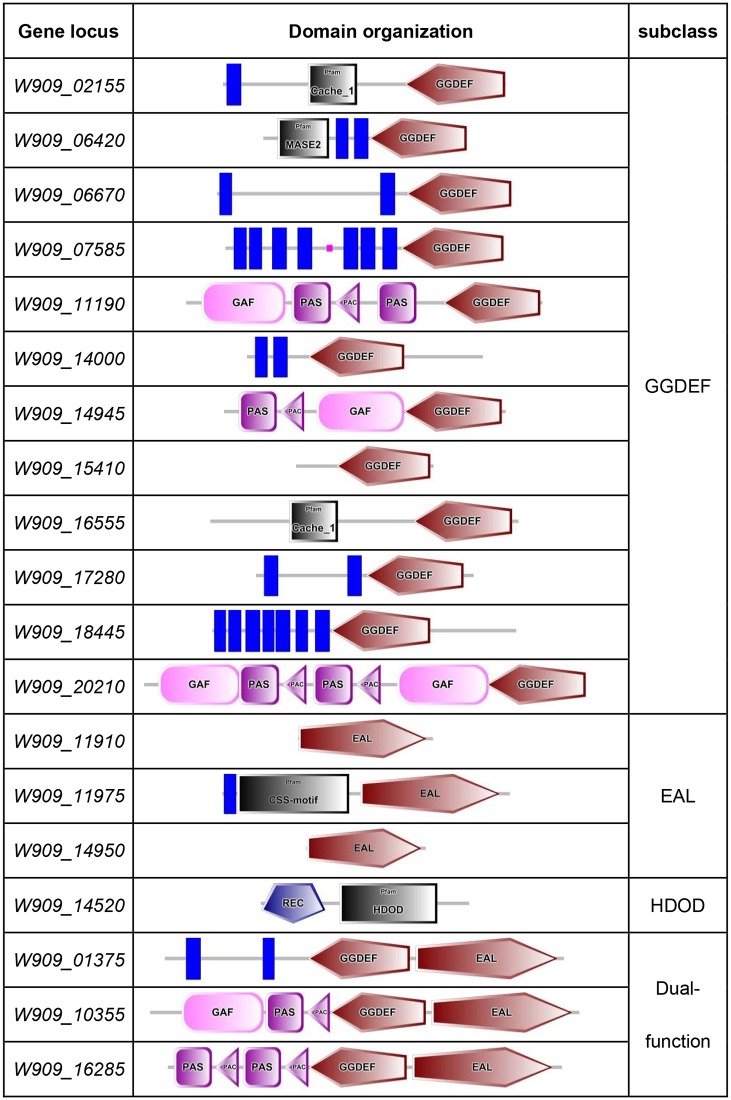
Predicted Domain organizations of proteins involved in c-di-GMP turnover in *D*. *zeae* EC1. Predicted domains of each protein involved in c-di-GMP turnover were analyzed based on SMART algorithm. The function description of each domain referred to both the Pfam and SMART databases. The GGDEF domain is a diguanylate cyclase domain that synthesizes c-di-GMP from 2 GTP molecules; EAL is one of the phosphodiesterase domain, named after its conserved residues, which play a role in metal binding; HDOD indicates the HD-GYP domain, another phosphodiesterase domain that uses a novel tri-nuclear catalytic iron centre involved in c-di-GMP binding and catalysis; CACHE domain comes from Calcium channels and Chemotaxis receptors that involve in bacterial chemotaxis; MASE2 is a novel integral membrane sensory domain; the GAF domain is named after three proteins, which are, cGMP-specific phosphodiesterases, adenylyl cyclases and FhlA; PAS domain functions as a signal sensor and is often associated with PAC domain; REC domain contains a phosphoacceptor site and is always the component of the TCS system which is phosphorylated by histidine kinase homologues; Blue stripe represents the transmembrane domain involved in signal transduction.

By gene function prediction and protein sequence analysis, we identified 19 genes involved in c-di-GMP turnover in the genome of *D*. *zeae* EC1, these genes include 12 GGDEF domain proteins, 3 EAL domain proteins, 1 HD-GYP protein and 3 GGDEF-EAL hybrid proteins, which are referred as GGDEF-, EAL&HD-GYP- and GGDEF&EAL-subclass respectively for the convenience of discussion. By using the SMART algorithm, the predicted domains of these 19 proteins were presented and listed (Fig 1).

In addition to the GGDEF, EAL and HD-GYP domains, 16 of the DGC and PDE proteins contain signal transduction or transmembrane domain (TM), which may play roles in interaction and responding to various environmental cues. Only three proteins contain a single domain, i.e., *W909_11910*, *W909_14950* and *W909_15410* with EAL, EAL and GGDEF domain, respectively. These proteins were predicted to be located within the inner membrane because of lacking obvious TM or signal domains.

The remaining 16 genes encode proteins with multi-domains. Among them, 9 contain TM domains at the upstream of GGDEF, EAL or the dual domains, including *W909_01375*, *W909_02155*, *W909_06420*, *W909_06670*, *W909_07585*, *W909_11975*, *W909_14000*, *W909_17280*, and *W909_18445*. These enzymes are likely to be either membrane bound or exported membrane proteins.

The sensor domains associated with the c-di-GMP metabolic domains include the PAS/PAC, REC, GAF and CACHE domains. Among them, PAS/PAC domain is known to sense oxygen concentration, redox or light[34–36], which is found in proteins encoded by *W909_10355*, *W909_11190*, *W909_14945*, *W909_16285*, and *W909_20210*. Under normal circumstances, PAS domains are always associated with PAC domains[37], which are also true for the proteins characterized in this study.

The REC domain contains a phosphoacceptor site that is phosphorylated by histidine kinase homologs, acting as one component of the two-component system (TCS), which may respond to a wide variety of stimuli, including nutrients, cellular redox state, quorum sensing signals, or antibiotics[38]. This domain is only contained by the HD-GYP protein encoded by *W909_14520*.

The GAF domain, named and found in three proteins, including cGMP-specific phosphodiesterase, adenylyl cyclase, and FhlA, was shown to interact with cyclic nucleotide within bacterial cells[39, 40]. Interestingly, four proteins encoded by *W909_10355*, *W909_11190*, *W909_14945*, and *W909_20210*, contain a GAF domain and c-di-GMP turnover domains, as well as PAS/PAC domain, arranged in tandem, which may suggest overlapping signaling pathways that modulate the c-di-GMP homeostasis in *D*. *zeae* strain EC1.

CACHE is predicted to be an extracellular protein domain and plays a role in small-molecule recognition[41]. In strain EC1, this domain is only found in the N-terminal of GGDEF domain proteins, such as those encoded by *W909_02155* and *W909_16555*. In addition, the unknown function domain MASE2, which is often found adjacent to the GGDEF domain[42], was found in the protein encoded by *W909_06420*.

Taken together, the above *in silico* analysis unveils a diverse group of putative sensor domains associated with c-di-GMP metabolic enzymes. Such structural features suggest that c-di-GMP turnover in *D*. *zeae* EC1 may be influenced by various environmental cues, and may also reflect the unusual ability of this bacterial pathogen in responding and adaptation to different host plants and environmental conditions.

### Roles of c-di-GMP metabolism in the biofilm formation

Biofilms are complicated matrix including bacterial secretions and cells that adhere to biological or non-biological surfaces, conferring resistance to antibiotics and increasing bacterial capabilities to infect host and reproduction[43]. Given the known role that high c-di-GMP level mostly triggers bacterial biofilm formation, whereas at a low c-di-GMP level, bacteria are likely to become motile[15], we assessed the effect of mutation of c-di-GMP metabolic genes on the biofilm formation in strain EC1.

**Fig 2:**
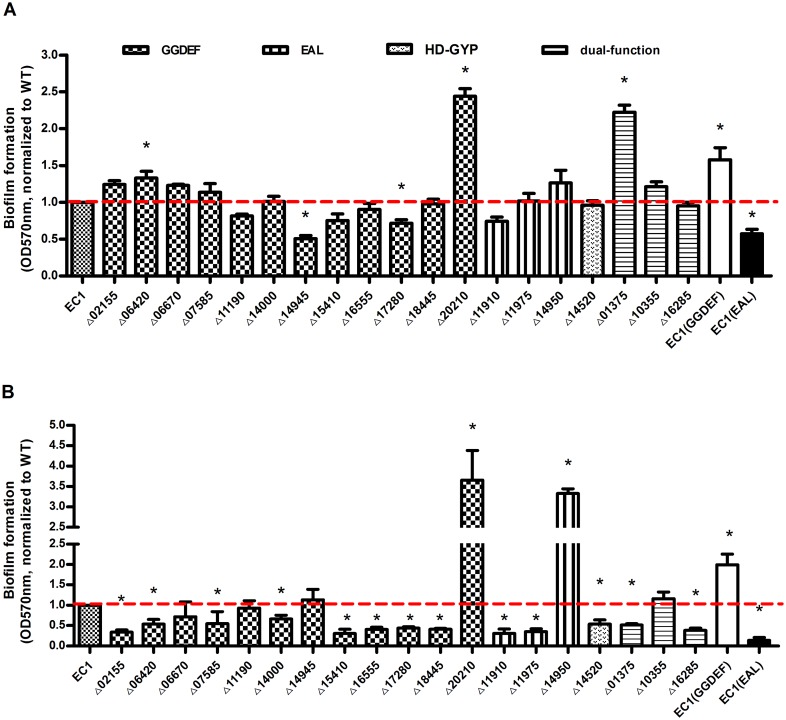
Biofilm formation assay. The shown is a measurement of biofilm formation quantification by *D*. *zeae* carrying mutations in genes across all the four subclasses of c-di-GMP turnover proteins in SOBG (A) and LB (B). One microliter of overnight strains was added to the medium in a ratio of 1:100 on a 96-well microplate, incubating at 28°C with 150 rpm shaking for 18 h. The biomass of biofilm attached cells was measured under optical density at 570 nm after staining with crystal violet. Dotted lines denoted the wild-type level, for easy comparison. Final results of each mutant were normalized to that of the wild-type EC1, which was set to a value of 1, for easy comparison. Experiments were repeated at least three times in triplicates. *, P < 0.05 (Student’s *t*-test).

Quantification of biofilm formation was conducted on two different media, the SOBG cell aggregation inducing medium and the LB medium (Fig 2). Yap *et al*. [10] found that cell aggregation behaviors of *Dickeya* strain Ech3937 could induce biofilms and pellicles in a rich medium, which is carbon source dependent. Therefore, we used SOB plus glycerol (SOBG) and common LB to measure solid surface-associated biofilms and further explore different changes between these two media in each mutant, which might indicate different signaling pathway to regulate biofilm formation through c-di-GMP pools in these two media.

In SOBG medium (Fig 2A), mutation of 5 genes significantly changed the biofilm formation by over 25% compared with the wild type EC1. The majority of mutants produced biofilm at a level similar to wild-type strain or moderately less. Intriguingly, in the GGDEF-only subclass, mutants showed differentiated results with raised biofilm formation in 5 mutants and decreased or similar level as wild type in the others. Mutation of *W909_14945* (Δ14945) and *W909_17280* (Δ17280) reduced biofilm formation by 49.18% and 28.48% respectively, compared with wild-type control; whereas mutation of *W909_20210* (Δ20210) and *W909_06420* (Δ06420) increased the biofilm formation by over 100% and by nearly 33% respectively. Mutation of most genes encoding EAL or HD-GYP domain did not significantly affect biofilm formation, except that deletion of *W909_01375* (Δ01375) increased biofilm formation by about 122.54%.

In LB medium, only 2 mutants, i.e., Δ14950 and Δ20210, produced significantly higher levels of biofilms than strain EC1, whereas at least 12 mutants showed drastically decreased biofilm formation (Fig 2B). Intriguingly, while mutation of some genes, such as *W909_20210* and *W909_14950*, resulted in similar trend of alternations in both SOBG and LB media, mutants of *W909_01375*, *W909_02155* and *W909_06420* showed a contrast pattern of changes in biofilm formation, i.e., increased biofilm formation in SOBG and decreased in LB (Fig 2A and 2B). Situation also happened that biofilm formation changed only in one medium, for example, mutants of *W909_14000*, *W909_16555*, *W909_18445*, *W909_11975*, *W909_14520* and *W909_16285*, showed greatly decreased biofilm production in LB medium (Fig 2B), but maintained a similar level as the wild-type strain EC1 in SOBG medium (Fig 2A).

As controls, we overexpressed the *wspR* and *rocR* genes from *P*. *aeruginosa* under the control of the *lac* promoter in *D*. *zeae* strain EC1 and tested their impact on bacterial biofilm formation. These two genes encode the GGDEF and EAL domain enzymes, respectively, and their enzymatic activity in synthesis and degradation of c-di-GMP has been demonstrated in previous studies[29, 30]. Results showed that overexpression of *wspR* encoding a GGDEF enzyme increased the biofilm formation in both SOBG and LB medium, whereas overexpression of the EAL domain gene *rocR* reduced this phenotype (Fig 2).

### Roles of c-di-GMP metabolism in flagella-mediated motile lifestyles

**Fig 3:**
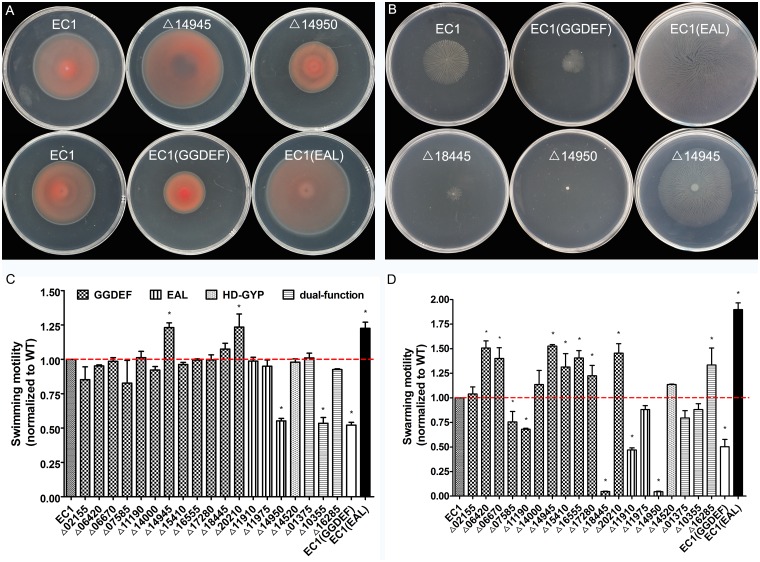
Flagella-mediated motile assay on c-di-GMP metabolism mutants of *D*. *zeae* EC1. Overexpression of GGDEF domain in EC1 reduced both the swimming and swarming motility while overexpression of EAL domain enhanced both significantly. A. Mutation of *W909_14945* enhanced swimming motility whereas mutation of *W909_14950* (Δ14950) reduced half swimming motility compared with wild-type EC1; B. Δ14950 abolished the swarming motility while Δ14945 enhanced; C. diameter of swimming zones was measured after 28 h inoculation. D. diameter of swarming zones was measured after 24 h inoculation. Dotted lines denoted the wild-type level, for easy comparison. Final results of each mutant in C and D were normalized to that of the wild-type EC1, which was set to a value of 1, for easy comparison. Experiments were repeated at least three times in triplicates. *, P < 0.05 (Student’s *t*-test).

Motile and sessile lifestyle transition mainly depends on flagella-mediated motility and biofilm formation. Most of these behaviors are associated with c-di-GMP concentration in bacteria[44–46]. Swimming and swarming motility are two surface translocation behaviors enabled by the action of bacterial flagella system. According to micromorphological pattern and the film of surface fluid, swimming is different from swarming in that the former is highly organized in micromorphological pattern with continuous and regular movement, and the latter is an unorganized and randomly moving action in thick surface fluid[47]. Here, we tested the swimming and swarming motility of the mutants to determine the roles of c-di-GMP metabolic enzymes in the modulation of bacterial motility (Fig 3).

Swimming motility was tested in semisolid medium, and the diameter of swimming zone was measured at 28 h post inoculation. Results showed that overexpression of the known GGDEF domain protein WspR inhibited the swimming ability of strain EC1, while overexpression of the EAL domain protein RocR generated a larger swimming zone than the parental strain EC1 (Fig 3A). Only 4 mutants, Δ14945, Δ20210, Δ14950 and Δ10355, showed significantly changed swimming motility by over 20% compared with wild type EC1 (Fig 3C). Among the 12 genes encoding only GGDEF domain proteins, only two mutants (Δ14945 and Δ20210) significantly enhanced swimming motility by more than 20% compared with EC1. Similarly, only Δ14950 out of the four PDE (EAL or HDOD domain enzymes) mutants, showed significantly decreased swimming motility by over 44% comparing with EC1. Results of the four GGDEF-EAL dual domain mutants were similar to that of the PDE mutants with only mutant Δ10355 showed significantly reduced swimming motility (53.3% of the wild-type level).

Swarming motility was assessed in swarming semisolid medium, and diameters of deep branches or tendril zones were measured (Fig 3D). Similar to *P*. *aeruginosa* PA14, wild type EC1 swarmed in dendritic shape[48]. As expected, overexpression of the GGDEF domain gene *wspR* inhibited the swarming motility of strain EC1, while overexpression of the EAL gene *rocR* in EC1 showed enhanced swarming motility (Fig 3B). Different from the results of swimming motility, nearly all the mutants were affected in swarming. In the GGDEF-only subclass, compared with wild-type strain EC1, swarming was increased by more than 40% in 5 mutants, such as Δ06420, Δ06670, Δ14945, Δ16555 and Δ20210, while reduced in mutants Δ07585, Δ11190 and Δ18445 (Fig 3D). In the EAL-only subclass, all 3 mutants showed decreased swarming motility, in particular, Δ14950 lost swarming motility by more than 95% (Fig 3B and 3D). Interestingly, mutation of HDOD domain raised swarming motility. In the last subclass, dual-function domain, mutants of Δ01375, and Δ10355 reduced swarming, while Δ16285 swarmed about 33.5% longer than wild type EC1.

### Roles of c-di-GMP metabolism genes in regulating exoenzymes production

**Fig 4:**
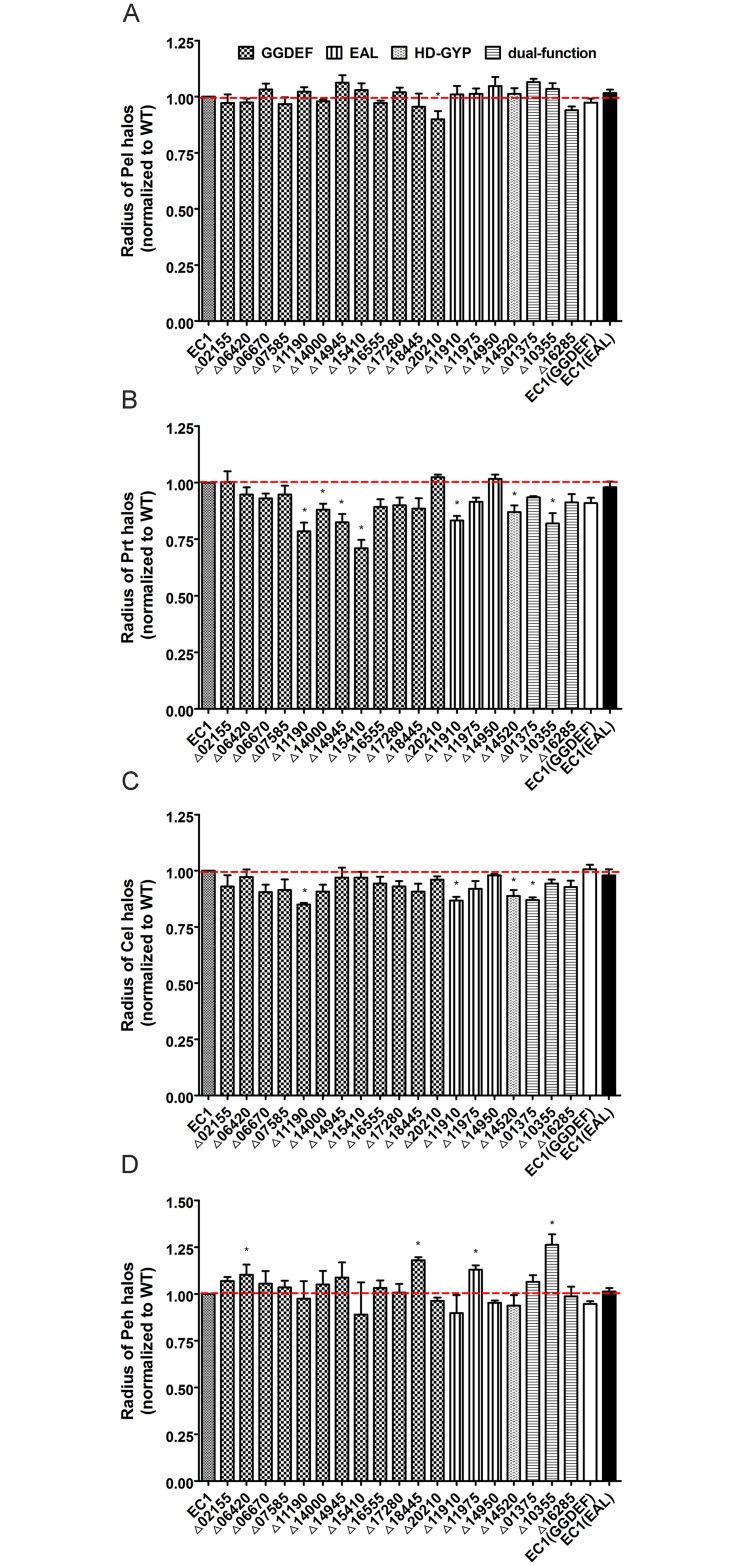
Exoenzyme production of *D*. *zeae* mutants of c-di-GMP turnover proteins. Strains were cultured in LB medium until OD_600_ value reached about 1.5. Production of Pel (A), Prt (B), Cel (C), and Peh (D) was measured in specific media with their own substrates and visualized with particular methods. For semi-quantification of enzyme production, radium of each halo was measured. Dotted lines denoted the wild-type level, for easy comparison. Final results of each mutant were normalized to that of the wild-type EC1, which was set to a value of 1, for easy comparison. Experiments were repeated at least three times in triplicates. *, P < 0.05 (Student’s *t*-test).

Pathogenic bacteria can produce cell wall degrading enzymes (exoenzyme) to infect host plants and induce disease development. Exoenzymes including Pel, Prt, Cel and their isoenzymes are known as the critical virulence factors in *D*. *dadantii* 3937[49, 50]. As a global regulating system in bacteria, c-di-GMP signaling was identified to regulate virulence in many bacteria. In the most destructive crucifer phytopathogen, *Xanthomonas campestris* cv. *campestris*, c-di-GMP degradation enzymes positively control the synthesis of extracellular enzymes, including endoglucanase and endomannanase[31, 51]. In this study, we measured 4 types of exoenzymes production in specific media, including Pel, Prt, Cel and Peh (Fig 4).

In Pel assay (Fig 4A), most of EC1 derivatives generated no significant change, only Δ20210 showed reduced Pel production by about 10%. It seems that c-di-GMP pathway makes no significant influence on Pel production in *D*. *zeae* strain EC1, which is supported by the results of overexpression of constitutively expressed known GGDEF and EAL proteins (Fig 4A).

In Prt assay (Fig 4B), nearly all mutants (16/19) showed decreased protease production, amongst, 7 with significantly reduced Prt production. In the GGDEF-subclass, Prt production of Δ15410 and Δ11190 was reduced by 28.93% and 21.33%, respectively, compared with the wild type. Another 2 mutants Δ14000 and Δ14945, reduced the enzyme production by 11.79% and 17.35%, respectively. Mutation of the genes in EAL-subclass also resulted in reduced Prt production. Mutant Δ11910 reduced protease production by 16.60%. The HDOD mutant Δ14520 produced only about 85% proteases compared with that of wild-type EC1. In the dual-function subclass, all mutants showed reduced Prt production.

In Cel assay (Fig 4C), the amount of enzymes produced by most mutants was comparable with that by EC1. However, 4 mutants showed decreased production of cellulases, i.e., Δ11190, Δ11910, Δ14520 and Δ01375 produced fewer cellulases by 15.03%, 12.98%, 11.07%, and 13.30%, respectively.

Different from the Prt plate assay, Peh assay result showed that the majority of mutants (11/19) displayed increased Peh production (Fig 4D). Nevertheless, only 4 showed a significant increase by above 10%. In the GGDEF containing only subset, Δ06420 and Δ18445 produced more 10.33% and 18.39% of Peh respectively. The only EAL deletion mutant, Δ11975, increased Peh secretion at nearly same level (about 13%). Among the last subset of dual domain proteins, Δ10355 produced increased enzymes by 26.24%.

### Effect of c-di-GMP on zeamine production

**Fig 5:**
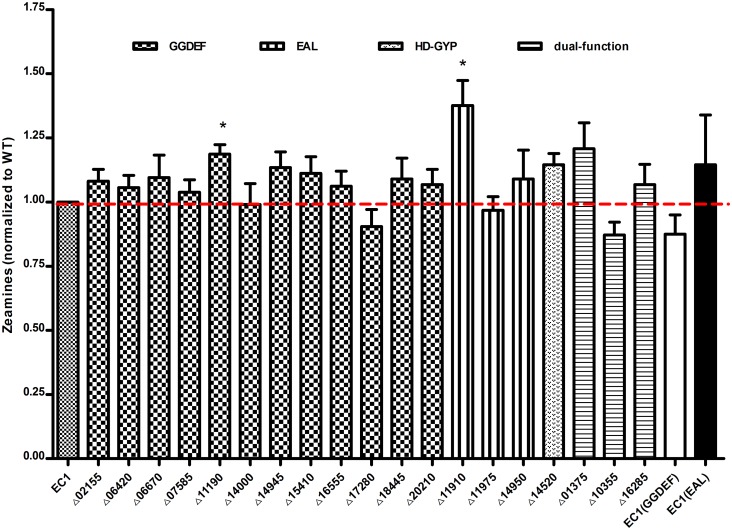
Zeamine production of *D*. *zeae* c-di-GMP mutants. The antibiotic activity was measured in 20 mL of LB agar medium, overlaid with 5 mL of 1% agarose containing 1.0 × 10^8^ cells of fresh *E*. *coli* DH5α after solidification. Radium of inhibition zone surrounding each well was measured and transformed into toxin units according to the formula: zeamines (units) = 0.5484^e0.886x^, (R^2^ = 0.9957), X is the width in millimeters of the growth inhibition zone surrounding each well. Dotted lines denoted the wild-type level, for easy comparison. Final results of each mutant were normalized to that of the wild-type EC1, which was set to a value of 1, for easy comparison. Experiments were repeated at least three times in triplicates. *, P < 0.05 (Student’s *t*-test).

Zeamines display potent antibiotic effect, and can inhibit and kill both gram-positive and gram-negative bacteria[12]. Intriguingly, the concentration of the c-di-GMP molecule in the bacterial cell was reported to affect the virulence of certain strains[15]. Thus, exploring the impact of c-di-GMP turnover genes on zeamine production is particularly interesting. Given the antibiotic activity, we tested the production of zeamines by measuring the growth inhibition zones against *E*.*coli*. The widths of inhibition zones were measured and converted to zeamines toxin units using the formula described previously[13, 27]. In the optimized zeamines inducing medium LS5[27], deletion of genes encoding c-di-GMP metabolism resulted in minor changes in most cases, except mutants of *W909_11190* (Δ11190) and *W909_11910* (Δ11910) which showed significantly increased production of zeamines by about 19% and 37% (Fig 5). In contrast, overexpression of the known GGDEF or EAL genes in strain EC1 did not significantly affect the production of zeamines.

### Complementation analysis of two c-di-GMP turnover mutants

**Fig 6:**
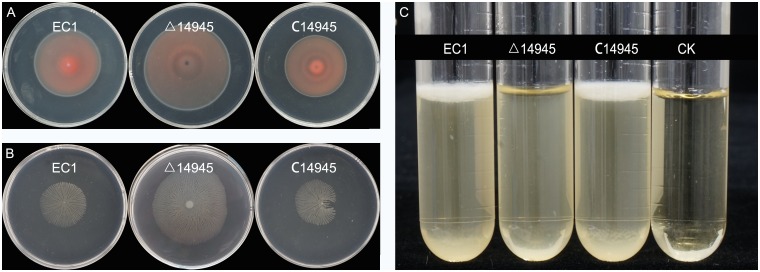
Swimming (A), swarming (B) and biofilm formation (C) of wild-type EC1, Δ14945 and the complementary strain C14945.

**Fig 7:**
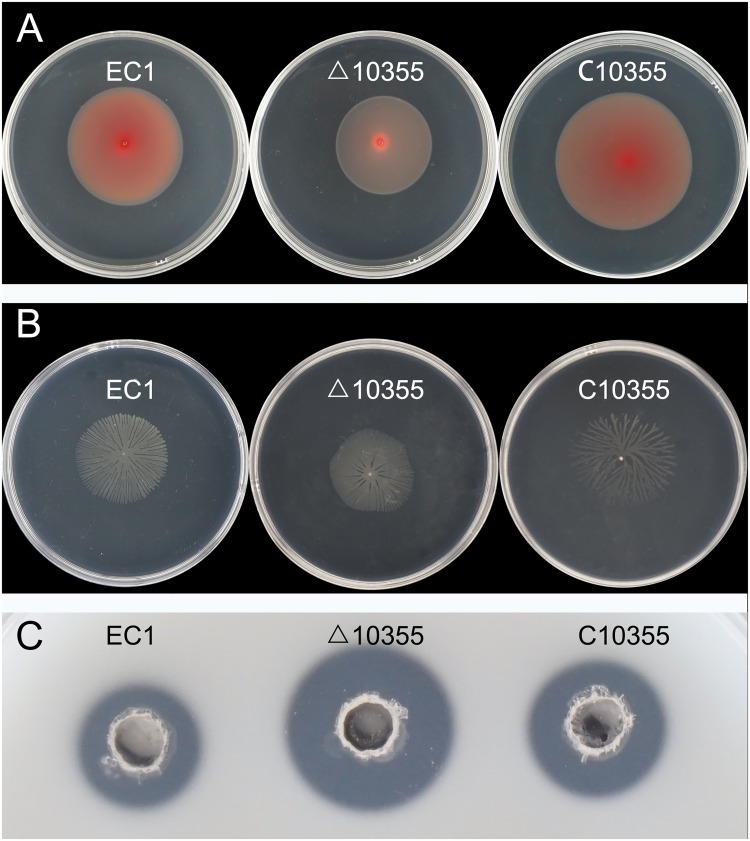
Swimming (A), swarming (B) and Peh production (C) of wild-type EC1, Δ10355 and the complementary strain C10355.

To confirm the validity of the knock-out mutants, two mutants with distinct phenotypes compared with wild-type EC1 were chosen for complementary analysis. The previous results showed that deletion of genes *W909_14945* and *W909_10355* caused significant changes in bacterial motility, biofilm formation, and exoenzyme production. Complementation of the mutant Δ14945 was performed by transferring the expression plasmid pBBR1-MCS4 fused with the complete ORF of *W909_14945* into the mutant. Phenotypes of complementary strain C14945 restored both swimming and swarming motility to wild type EC1 level, as well as biofilm formation (Fig 6). Another complementary strain C10355 restored Peh production and flagellar-based motility to EC1 level (Fig 7). Altogether, the above results confirmed the roles of c-di-GMP turnover genes in virulence modulation.

## Discussion

In the article, in-frame deletion mutants of all the c-di-GMP metabolic proteins in EC1 was constructed. Major phenotypes including biofilm formation, swimming, swarming, exoenzymes as well as zeamine production were assessed. Obvious differences in phenotypes of two mutants on a DGC and a PDE-encoding genes were restored by *in trans* complementation analysis.

In summary, overexpression of the GGDEF protein increased biofilm formation with poorer motility, while overexpression of the EAL protein showed contrary results in these two phenotypes. However, the two derivatives showed no differences on the production of exoenzyme and toxin zeamines. In the cell aggregate medium SOBG, the level of biofilm formation was higher than that in LB medium, which confirmed that cell aggregation promotes biofilm formation with many same characteristics[10]. All these phenotypic changes are consistent with the results of the GGDEF- and EAL- subclasses, except for Δ20210, Δ11910 and Δ11975. For example, deletion of GGDEF reduced biofilm formation in SOBG except Δ20210, Δ11910 and Δ11975 displayed dispersed phenotype on biofilm formation in contrast with the hyper biofilm mutant Δ14950 in EAL-subclass (Fig 2). Most GGDEF mutants increased swarming motility except Δ07585, Δ11190 and Δ18445, and all EAL mutants reduced motility, consistent with the EAL functions on motility (Fig 3). All the mutants produced similar level of exoenzymes and zeamines to EC1, except Δ11190 and Δ11910 produced more zeamines (Figs 4 and 5).

However, phenotypic contradiction was observed between overexpressed strains and subclass mutants. For example, deletion of *W909_20210* (GGDEF) formed hyper-biofilm, opposite to the GGDEF functions on biofilm formation. Deletion of *W909_11910* (EAL) dispersed biofilm, with diverse functions on biofilm formation compared with EAL domain overexpression in EC1. Several mutants showed significant changes in the production of exoenzymes and zeamines despite that either GGDEF or EAL overexpression strain showed similar production compared with EC1. One reason for the phenotypic differentiation is that the functional domains become ‘retired’ (i.e., lose their catalytic functions). Bioinformatics analysis and functional studies highlighted the existence of large numbers of enzymatically inactive GGDEF and EAL domain-containing proteins in bacterial strains, making that the GGDEF and EAL domain degenerated to become an important type of c-di-GMP effectors. One example of a catalytically incompetent GGDEF domain-containing protein is *P*. *aeruginosa* PelD, which contains an N-terminal GAF domain and a degenerate GGDEF domain in C-terminal[52]. X-ray crystal structure and isothermal titration calorimetry analysis determined that a RxxD motif is critical for this degenerate GGDEF domain binding dimeric c-di-GMP, which produces conformational change, regulates the production of PEL exopolysaccharide and biofilm formation[52, 53]. Sequence alignment of domain analysis in the protein encoded by *W909_11910* generated an abnormal ‘EQL’ motif, which showed high homology with *ydiV*, an inactive EAL family phosphodiesterase in *E*.*coli* K-12[54], predicting that *W909_11910* is degenerated. Another reason to explain this ‘loss of function’ phenomenon is the sequestration of c-di-GMP control modules, that is, these c-di-GMP turnover proteins may be functioning in parallel on different compartments within one cell, separating several individual c-di-GMP regulatory pools[55, 56]. Obviously, each compartment includes the entire c-di-GMP regulatory modules (sensor domain, turnover pool, and effector) and mostly the input signal of each compartment differs from each other, which bacteria held to survive and efficiently adapt to different surroundings[56]. In *E*. *coli*, different regulatory modules of c-di-GMP signaling pathway were identified, i.e., the YaiC (DGC)/YoaD (PDE) module regulates biofilm cellulose formation through c-di-GMP binding to BcsA[57]; another two c-di-GMP pools modulate flagella-mediated motility and curli adhesion, which synthesized and degraded by DGCs (YedQ, YegE, and YdaM) and 2 PDEs (YhjH and YciR)[44, 45, 55, 58]. The functional antagonism of each compartment is stimulated regularly at different times according to different environmental signals. As in EC1, Δ14945 reduced more than 40% biofilm in SOBG medium but remained wild type level in LB medium, indicating that different environment ingredients trigger various phenotypes of bacteria. The “enzymatic conundrum” also exists within each compartment which regularly involves two proteins with opposite enzymatic activities. One scenario is supposed that the enzymatic activities of both proteins are active and regulated differentially by environmental signals. A well-studied example of this “enzymatic conundrum” is the Dos system in *E*. *coli*, which contains the DosC (GDC)–DosP (PDE) complex in one c-di-GMP pool to modulate under the condition of the concentration of oxygen[59, 60]. Under anaerobic conditions, the c-di-GMP concentration of the Doc system pool is accumulated, where the DosC is activated and the DosP is inhibited[60].

Intriguingly, phenotypic contradiction happened among almost all subclass which means that opposite phenotypes generated within a subclass or similar results exist among two functional contrary subclasses, i.e., in the GGDEF subclass, Δ14945 reduced more than 40% biofilm in SOBG medium while mutation of *W909_20210* (Δ20210) formed hyper-biofilm, indicating that different function of the two genes in biofilm formation. Amino alignment in SMART revealed that both of genes *W909_14945* and *W909_20210* encoded a RxxD (where “x” is any residue) and SGDEF/GGEEF motif positioning five amino acids downstream, indicating they have the catalytic activity. RxxD is characterized as an allosteric inhibitory site (I site) in many GGDEF containing proteins and SGDEF was identified as a non-canonical c-di-GMP synthesis motif in the phytopathogen *Pectobacterium atrosepticum* SCRI1043 (Pba1043)[15, 61, 62]. The phenotype of a DGC gene knocking out mutant was supposed to generate similar output as overexpressing a PDE domain containing gene, which Δ14945 showed similar phenotype as an EAL overexpression strain did. Contrary output of Δ20210 displayed only in biofilm, which demonstrated Δ20210 boosted bacterial biofilm possibly without increasing the c-di-GMP concentration through activating the I site[63], which possess as a c-di-GMP receptor to prevent overproduction of c-di-GMP in the cell, blocking the functional GGEEF domain.

As a model strain in *Dickeya* genus, the genome sequence of *D*. *dadantii* 3937 was assembled completely and released in 2007[64]. A total of 18 genes with predicted GGDEF and EAL domains were identified in *D*. *dadantii* genome, including 12 genes with GGDEF-subclass domain, four with EAL-subclass domain and 2 with dual-function domains (ASAP website; https://asap.ahabs.wisc.edu/asap/home.php). Based on the genome sequence released recently[26], we predicted same number of c-di-GMP turnover genes in *D*. *zeae* EC1, except that the one (*W909_14520*) belongs to the HD domain superfamily[65]. Sequence analysis showed that the amino acid homology of 14 genes was higher than 70% between EC1 and 3937, and 4 genes (*W909_02155*, *W909_14000*, *W909_16285* and *W909_18445*) could not be found homologous in 3937 (S4 Table). The remaining putative HD-GYP domain encoding gene *W909_14520* was found highly conserved with the homolog in *D*. *dadantii* 3937 (S4 Table). The high homology of the c-di-GMP genes in these two strains indicated the c-di-GMP similarity in evolutionary history. Being an important phytopathogen in *Dickeya* genus, *D*. *dadantii* 3937 was thoughtfully studied for over 30 years[66], revealing the bacterial pathogenesis on hosts and various virulence factors, including exoenzymes, iron transport, indigodine pigment and extracellular polysaccharide catabolism[50, 67–70]. Recent studies on pathogen’s response to plant defense mainly include secretion system, flagellar motility, and biofilm formation[25, 71]. Given that the critical functions as virulence factors that have been reported in *D*. *dadantii* 3937, we focused on 4 genes except for *gcpA* in *D*. *zeae* EC1 (S4 Table) to compare the phenotypic changes after in-frame deletion, including biofilm formation, swimming motility, swarming motility, and Pel production[25]. Δ06420 and its homologous gene mutant ΔgcpC showed wild-type background in all the tested factors except for swarming motility was increased by 50.72% in Δ06420. In *D*. *dadantii* 3937, ΔgcpD displayed no significant changes, and the deletion of its homologous gene *W909_11910* in EC1 showed similar phenotypes in Pel production and swimming motility, but reduced in biofilm formation and swarming motility. Interestingly, Δ10355 and Δ14950 showed partially similar phenotype with mutants of their highly homologous genes. Δ10355 lost nearly half ability in swimming motility and reduced by 12.07% in swarming motility, the same tendency as ΔecpB which swam less than 60% of the wild-type; however, biofilm formation is enhanced by 5-fold and Pel production is decreased by 50% in ΔecpB, but no difference was observed in Δ10355. Another mutant Δ14950 displayed the similar trend in biofilm formation and flagella-mediated motility, as well as ΔecpC. In the assay of Pel production, mutation of *W909_14950* showed wild-type phenotype while ΔecpC produced less than 50% of Pel.

Taken together, our research revealed the complicated relationships between the c-di-GMP signaling pathway and various phenotypes, indicating the complex regulation networks of the c-di-GMP system in EC1. To our knowledge, c-di-GMP signaling pathway seems to primarily modulate the motility-to-sessility transition lifestyle in EC1. The second messenger c-di-GMP was reported to generate strong influence on this lifestyle transition[72–75]. As a model strain, *P*. *aeruginosa* is an important opportunistic human pathogen that causes cystic fibrosis through acute or chronic infections, which relies on motile to sessile transition[21, 76]. Biofilm formation is critical for bacteria to resist the host’s immune defenses and antibiotic therapy, which make many infections hard to be resolved. Given that bacterial lifestyle transition is critical for virulence, mutants with significant difference in biofilm and motility were chosen for testing the pathogenicity on potato tubers. Soft rot symptoms of all tested mutants were slightly attenuated compared with wild type (S3 Fig). To our knowledge, multiple c-di-GMP turnover proteins are usually encoded in one bacterial genome, a single mutation of the c-di-GMP turnover genes is not enough to explore the practical function of c-di-GMP regulation on both virulence traits and bacterial virulence, methods of complete deletion of all DGC or PDE proteins remain to be done to totally shut down the c-di-GMP turnover, the signal network connecting the environmental signal and the output phenotypes remained to be further studied. Looking forward, in this article, both the methods and mutant strains can be possibly used as a helpful tool for exploring the role of the c-di-GMP molecule in EC1, and its regulation pathways as well.

## Supporting Information

S1 Fig Growth curves of EC1 and its derivatives. The growth curves of EC1 and derivatives were tested in SOBG (A), LB (B) and LS5 (C) media using 96-well polypropylene microliter dishes. Overnight bacterial cultures of each strain were transferred to the three media respectively. A. The majority of strains reached the stationary phase at 14 hr with OD_600_ value about 1.2 and turned into the decline phase at 24 hr. Considering the influence of biofilm formation in measuring the growth condition, the OD_600_ readings in the growth curve are reduced in Δ14945. B. Strains showed the uniformly weak growth curves in LB medium in which OD_600_ value failed to reach 0.4 in 30 hr without obvious stationary phase, except Δ14950 raised the OD_600_ value above to 0.6 and then reduced to about 0.4. C. Growth curves in LS5 medium showed the similar tendency of all strains, which increased sharply during 8 to 14 hr and reached stationary phase at 26 hr with OD_600_ value about 0.8 and reduced to 0.6 at 30 hr.
(TIF)

S2 Fig Gene amplification region of WspR and RocR in *P*. *aeruginosa*. (TIF)

S3 Fig Pathogenicity tests of c-di-GMP mutants on potato tubers. After being washed with sterile water and 70% ethanol throughout the surface, potato tubers were sliced to about 5 mm thickness and then each slice was placed onto two Whatman paper no. 3 filter papers (moistened with sterilized water) in a petri dish. The plant tissues were inoculated with 2 μl of bacterial cells at OD_600_ = 1.2 in the middle. Photographs were taken 24 h after incubation at 28°C. LB medium was set as the negative control.
(TIF)

S1 Table Strains used in this study. (DOCX)

S2 Table Plasmids used in this study. (DOCX)

S3 Table Primers used in this study. (DOCX)

S4 Table Homologous comparison of c-di-GMP metabolism proteins between *D*. *zeae* EC1 and *D*. *dadantii* 3937. (DOCX)
