# Supplementary figures and images for: Genetic Modulation of c-di-GMP Turnover Affects Multiple Virulence Traits and Bacterial Virulence in Rice Pathogen Dickeya zeae
Source: PLoS One. 2016 Nov 17;11(11):e0165979. doi: 10.1371/journal.pone.0165979 (PMC5113947; doi:10.1371/journal.pone.0165979)

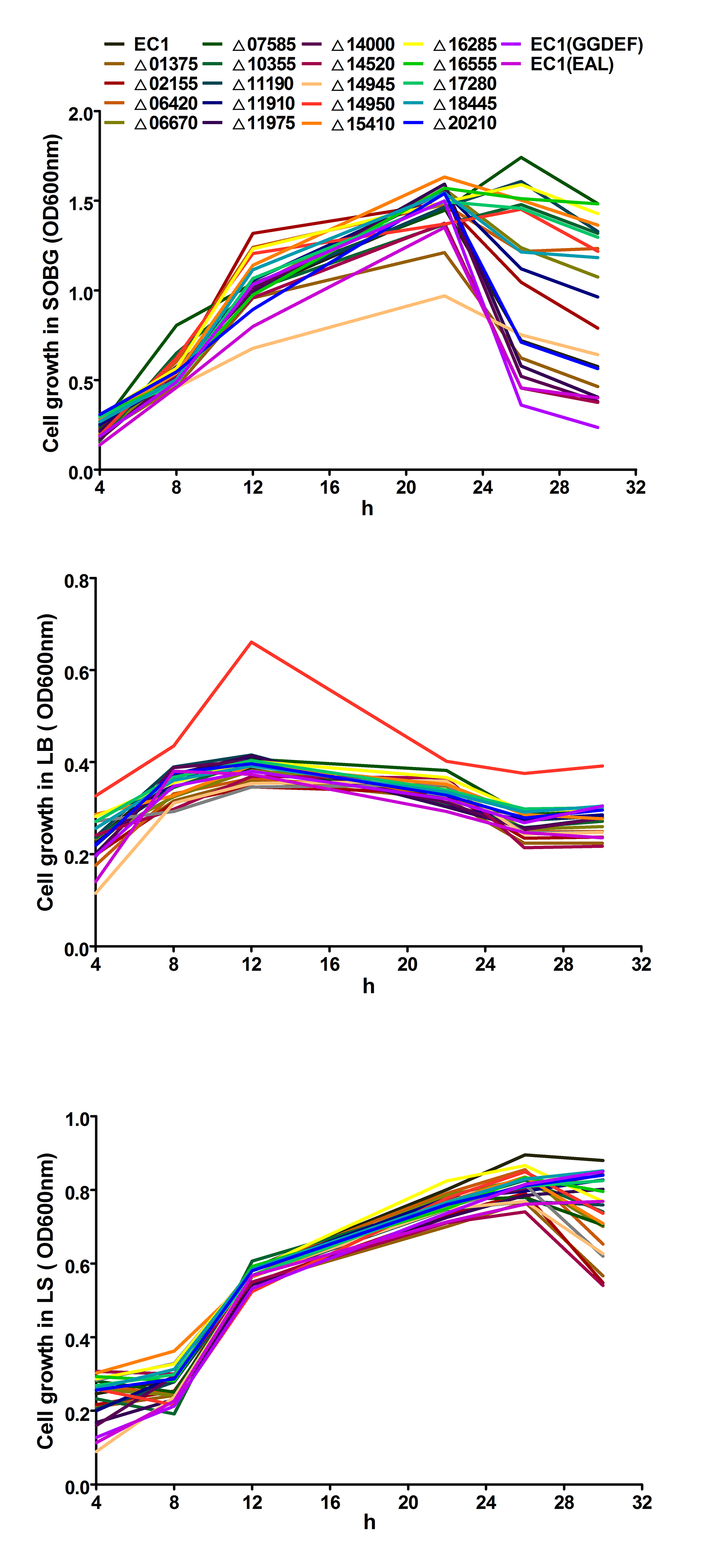

Supplement: S1 Fig — The growth curves of EC1 and derivatives were tested in SOBG (A), LB (B) and LS5 (C) media using 96-well polypropylene microliter dishes. Overnight bacterial cultures of each strain were transferred to the three media respectively. A. The majority of strains reached the stationary phase at 14 hr with OD600 value about 1.2 and turned into the decline phase at 24 hr. Considering the influence of biofilm formation in measuring the growth condition, the OD600 readings in the growth curve are reduced in Δ14945. B. Strains showed the uniformly weak growth curves in LB medium in which OD600 value failed to reach 0.4 in 30 hr without obvious stationary phase, except Δ14950 raised the OD600 value above to 0.6 and then reduced to about 0.4. C. Growth curves in LS5 medium showed the similar tendency of all strains, which increased sharply during 8 to 14 hr and reached stationary phase at 26 hr with OD600 value about 0.8 and reduced to 0.6 at 30 hr. (TIF) [file pone.0165979.s001.tif]

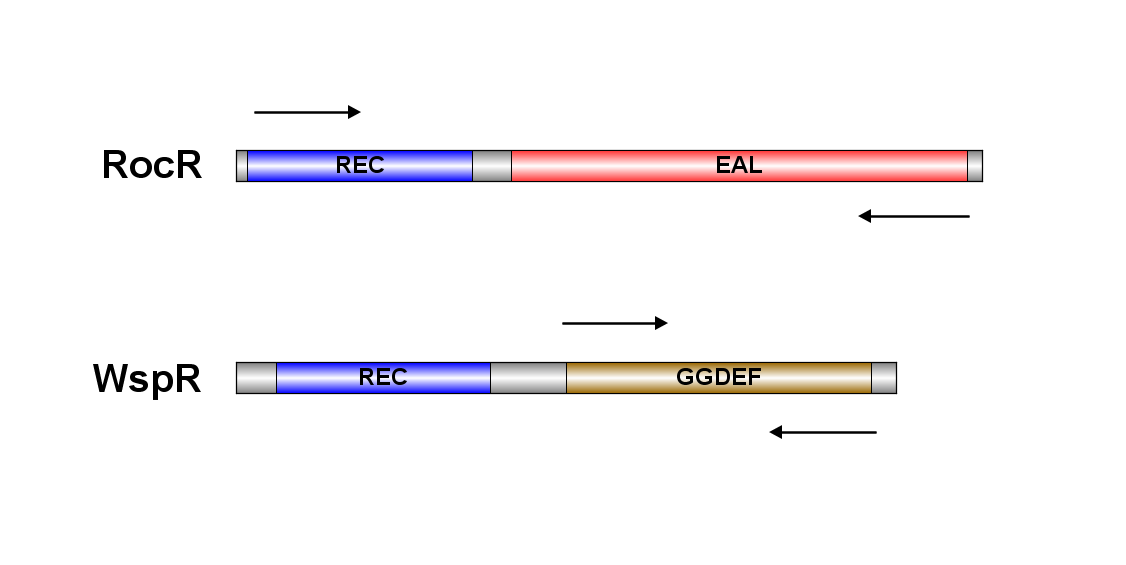

Supplement: S2 Fig — (TIF) [file pone.0165979.s002.tif]

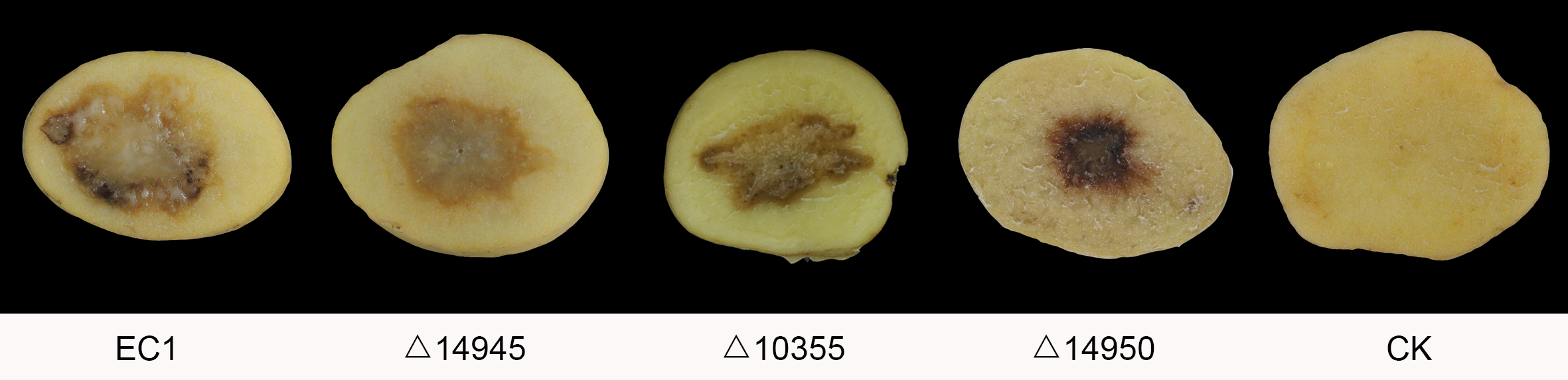

Supplement: S3 Fig — After being washed with sterile water and 70% ethanol throughout the surface, potato tubers were sliced to about 5 mm thickness and then each slice was placed onto two Whatman paper no. 3 filter papers (moistened with sterilized water) in a petri dish. The plant tissues were inoculated with 2 μl of bacterial cells at OD600 = 1.2 in the middle. Photographs were taken 24 h after incubation at 28°C. LB medium was set as the negative control. (TIF) [file pone.0165979.s003.tif]
