# Supplementary material for: Genetic Modulation of c-di-GMP Turnover Affects Multiple Virulence Traits and Bacterial Virulence in Rice Pathogen Dickeya zeae
Source: PLoS One. 2016 Nov 17;11(11):e0165979. doi: 10.1371/journal.pone.0165979 (PMC5113947; doi:10.1371/journal.pone.0165979)
Supplement: S1 Table — (DOCX) [file pone.0165979.s004.docx]

**S1** **Table. Strains used in this study.**

| **Strains** | **Relevant phenotype** | **References of source** |
| --- | --- | --- |
| EC1 | Wild-type *D. zeae* EC1, Pm^r^ | Lab collection |
| △01375 | *W909_01375* knock-out mutant of EC1 | This study |
| △02155 | *W909_02155* knock-out mutant of EC1 | This study |
| △06420 | *W909_06420* knock-out mutant of EC1 | This study |
| △06670 | *W909_06670* knock-out mutant of EC1 | This study |
| △07585 | *W909_07585* knock-out mutant of EC1 | This study |
| △10355 | *W909_10355* knock-out mutant of EC1 | This study |
| △11190 | *W909_11190* knock-out mutant of EC1 | This study |
| △11910 | *W909_11910* knock-out mutant of EC1 | This study |
| △11975 | *W909_11975* knock-out mutant of EC1 | This study |
| △14000 | *W909_14000* knock-out mutant of EC1 | This study |
| △14520 | *W909_14520* knock-out mutant of EC1 | This study |
| △14945 | *W909_14945* knock-out mutant of EC1 | This study |
| △14950 | *W909_14950* knock-out mutant of EC1 | This study |
| △15410 | *W909_15410* knock-out mutant of EC1 | This study |
| △16285 | *W909_16285* knock-out mutant of EC1 | This study |
| △16555 | *W909_16555* knock-out mutant of EC1 | This study |
| △17280 | *W909_17280* knock-out mutant of EC1 | This study |
| △18445 | *W909_18445* knock-out mutant of EC1 | This study |
| △20210 | *W909_20210* knock-out mutant of EC1 | This study |
| C10355 | △10355 containing *W909_10355* coding region at the downstream of *lac* promoter, Ap^r^ | This study |
| C14945 | △14945 containing *W909_14945* coding region at the downstream of *lac* promoter, Ap^r^ | This study |
| EC1(GGDEF) | EC1 containing a GGDEF domain coding region from *wspR* in PAO1 at the downstream of *lac* promoter, Ap^r^ | This study |
| EC1(EAL) | EC1 containing an EAL domain coding region from *rocR* in PAO1 at the downstream of *lac* promoter, Ap^r^ | This study |
| *E. coli* K12 CC118 | *gyrA*，*recA*，*λ pir* | Lab collection |
| *E. coli* DH5α | *deoR*, *recA*, *endA*, *hsdR*, *supE*, *thi*, *gyrA*, *relA* | Lab collection |
