# Supplementary material for: Genetic Modulation of c-di-GMP Turnover Affects Multiple Virulence Traits and Bacterial Virulence in Rice Pathogen Dickeya zeae
Source: PLoS One. 2016 Nov 17;11(11):e0165979. doi: 10.1371/journal.pone.0165979 (PMC5113947; doi:10.1371/journal.pone.0165979)
Supplement: S2 Table — (DOCX) [file pone.0165979.s005.docx]

**S2 Table. Plasmids used in this study.**

| **Plasmids** | **Relevant phenotype** | **References of source** |
| --- | --- | --- |
| pKNG101 | Suicide vector; Str^r^, *SacB*, *mobRK2*, *oriR6K* (pir-minus) | Lab collection |
| pRK2013 | Tra^+^, Mob^-^, ColE1-replicon, Kan^r^, Spe^r^ | Lab collection |
| pKNG-01375 | *W909_01375* knock-out fragment ligated on pKNG101 | This study |
| pKNG-02155 | *W909_02155* knock-out fragment ligated on pKNG101 | This study |
| pKNG-06420 | *W909_06420* knock-out fragment ligated on pKNG101 | This study |
| pKNG-06670 | *W909_06670* knock-out fragment ligated on pKNG101 | This study |
| pKNG-07585 | *W909_07585* knock-out fragment ligated on pKNG101 | This study |
| pKNG-10355 | *W909_10355* knock-out fragment ligated on pKNG101 | This study |
| pKNG-11190 | *W909_11190* knock-out fragment ligated on pKNG101 | This study |
| pKNG-11910 | *W909_11910* knock-out fragment ligated on pKNG101 | This study |
| pKNG-11975 | *W909_11975* knock-out fragment ligated on pKNG101 | This study |
| pKNG-14000 | *W909_14000* knock-out fragment ligated on pKNG101 | This study |
| pKNG-14520 | *W909_14520* knock-out fragment ligated on pKNG101 | This study |
| pKNG-14945 | *W909_14945* knock-out fragment ligated on pKNG101 | This study |
| pKNG-14950 | *W909_14950* knock-out fragment ligated on pKNG101 | This study |
| pKNG-15410 | *W909_15410* knock-out fragment ligated on pKNG101 | This study |
| pKNG-16285 | *W909_16285* knock-out fragment ligated on pKNG101 | This study |
| pKNG-16555 | *W909_16555* knock-out fragment ligated on pKNG101 | This study |
| pKNG-17280 | *W909_17280* knock-out fragment ligated on pKNG101 | This study |
| pKNG-18445 | *W909_18445* knock-out fragment ligated on pKNG101 | This study |
| pKNG-20210 | *W909_20210* knock-out fragment ligated on pKNG101 | This study |
| pBBR1-MCS4 | Expression vector contains a *lac*Z promoter, Ap^r^ | Lab collection |
| pBBR1-10355 | pBBR1-MCS4 carries the coding region of *W909_10355* at down-stream of *lac* promoter, Ap^r^ | This study |
| pBBR1-14945 | pBBR1-MCS4 carries the coding region of *W909_14945* at down-stream of *lac* promoter, Ap^r^ | This study |
| pBBR1-GGDEF | pBBR1-MCS4 carries the coding region of *wspR* at down-stream of *lac* promoter, Ap^r^ | This study |
| pBBR1-EAL | pBBR1-MCS4 carries the coding region of *rocR* at down-stream of *lac* promoter, Ap^r^ | This study |
