# Supplementary material for: Genetic Modulation of c-di-GMP Turnover Affects Multiple Virulence Traits and Bacterial Virulence in Rice Pathogen Dickeya zeae
Source: PLoS One. 2016 Nov 17;11(11):e0165979. doi: 10.1371/journal.pone.0165979 (PMC5113947; doi:10.1371/journal.pone.0165979)
Supplement: S3 Table — (DOCX) [file pone.0165979.s006.docx]

**S3 Table. Primers used in this study.**

| **Primer name** | **Primer sequence (5’-3’; underlined letter represents cleavage sites)** | **Amplicon** |
| --- | --- | --- |
| W909_01375-1 | cgggatccAATGATTTTCACCCACGC | *W909_01375* deletion |
| W909_01375-2 | CGGACACTGGAGTAAGGTTTTCGTCAGCAGCGTCATTACT |  |
| W909_01375-3 | AGTAATGACGCTGCTGACGAAAACCTTACTCCAGTGTCCG |  |
| W909_01375-4 | ggactagtAATTGCACGAACCACCGACA |  |
| W909_02155-1 | cgggatccGTATTGGAACCTGGTGATGA | *W909_02155* deletion |
| W909_02155-2 | AGATGGTCGATGCTTTCACCTTCATTGGACAGCACCCAGT |  |
| W909_02155-3 | ACTGGGTGCTGTCCAATGAAGGTGAAAGCATCGACCATCT |  |
| W909_02155-4 | ggactagtGGCTGAGACAGTACGCATAA |  |
| W909_06420-1 | cgggatccCGTTGATAGTGAGGTGAATC | *W909_06420* deletion |
| W909_06420-2 | TCTGGAACAAGCACGACGCAGGAACATAAACACGTCTGGC |  |
| W909_06420-3 | GCCAGACGTGTTTATGTTCCTGCGTCGTGCTTGTTCCAGA |  |
| W909_06420-4 | ggactagtAAGTGTTGCTACGCTGTCAG |  |
| W909_06670-1 | cgggatccACAGCTATCATTGTTGGG | *W909_06670* deletion |
| W909_06670-2 | CTTACCGTCACCACCTGTTCCGAGTTCCAGAAATTCCAGG |  |
| W909_06670-3 | CCTGGAATTTCTGGAACTCGGAACAGGTGGTGACGGTAAG |  |
| W909_06670-4 | ggactagtCTTCAGGTAGCTGGCAATCA |  |
| W909_07585-1 | cgggatccGTGTCGTCGTGCTGACTCAA | *W909_07585* deletion |
| W909_07585-2 | CACGTGCTGTTGGTTGAACAAGCCGTAGAACATCGTATCT |  |
| W909_07585-3 | AGATACGATGTTCTACGGCTTGTTCAACCAACAGCACGTG |  |
| W909_07585-4 | ggactagtGAATGATGGCACGGTTTTCC |  |
| W909_10355-1 | cgggatccTGATGCTTCATGAGGCGATG | *W909_10355* deletion |
| W909_10355-2 | ATTCGCAATTCACTGACAGGCCCTCTTTACTCTGTCTCTT |  |
| W909_10355-3 | AAGAGACAGAGTAAAGAGGGCCTGTCAGTGAATTGCGAAT |  |
| W909_10355-4 | ggactagtCCATGATAATCAAGCCTGCC |  |
| W909_11190-1 | cgggatccTGCAAGGCGAAAAACTCTCG | *W909_11190* deletion |
| W909_11190-2 | GAGGTAAATCGCCATCAGACACCTGATGATAGTGGCTGTC |  |
| W909_11190-3 | GACAGCCACTATCATCAGGTGTCTGATGGCGATTTACCTC |  |
| W909_11190-4 | ggactagtCATAATCATACGTGCGATGG |  |
| W909_11910-1 | cgggatccTTTGATGTGGAGCGAGGTGT | *W909_11910* deletion |
| W909_11910-2 | TCACCTGACTGAGAATAGCGCATAGTCAACTGCAAGCTGG |  |
| W909_11910-3 | CCAGCTTGCAGTTGACTATGCGCTATTCTCAGTCAGGTGA |  |
| W909_11910-4 | ggactagtAAGTGTTGCTACGCTGTCAG |  |
| W909_11975-1 | cgggatccTTACAACGCGACCCGAAAGT | *W909_11975* deletion |
| W909_11975-2 | TGTGTTGTCAGGGATTCTGCACTCATCCATCCGTGGTCTT |  |
| W909_11975-3 | AAGACCACGGATGGATGAGTGCAGAATCCCTGACAACACA |  |
| W909_11975-4 | ggactagtGCAGACCAGATCTTCGAACA |  |
| W909_14000-1 | cgggatccCCGATAATCATCCACTGGTC | *W909_14000* deletion |
| W909_14000-2 | TGATGTTCAGCTCTGTCTGGGATTCCAGCCATCCACATAG |  |
| W909_14000-3 | CTATGTGGATGGCTGGAATCCCAGACAGAGCTGAACATCA |  |
| W909_14000-4 | ggactagtCTACTGTTTGTGATGGTGGG |  |
| W909_14520-1 | cgggatccTTCTTCACGACCAAAGCCGT | *W909_14520* deletion |
| W909_14520-2 | CTTGTTCAGCACGCCAGTTTCTTCAGGTTCAGAGTAGCCA |  |
| W909_14520-3 | TGGCTACTCTGAACCTGAAGAAACTGGCGTGCTGAACAAG |  |
| W909_14520-4 | ggactagtAGCAACCATAGCGAGTACGT |  |
| W909_14945-1 | cgggatccAGGGAAACTGTGACGATAGC | *W909_14945* deletion |
| W909_14945-2 | TGGTAGATTTGGGTCTGGCTAATAACACTCTCCTCAGGCC |  |
| W909_14945-3 | GGCCTGAGGAGAGTGTTATTAGCCAGACCCAAATCTACCA |  |
| W909_14945-4 | ggactagtAATGAGCTGTCACTGGTCGT |  |
| W909_14950-1 | cgggatccCATCGAGATGGCTTGATTGG | *W909_14950* deletion |
| W909_14950-2 | TTATTGAGCCGGAGATAGGGTCAGCATCTGTAAGCAGTGC |  |
| W909_14950-3 | GCACTGCTTACAGATGCTGACCCTATCTCCGGCTCAATAA |  |
| W909_14950-4 | ggactagtATCGCTGTGTGCAGACCTAA |  |
| W909_15410-1 | cgggatccAGCCACTGCAGTTCACCTTT | *W909_15410* deletion |
| W909_15410-2 | GTTAGCTGGCAGTATCGATCTTACTCCAGAATGTGGGGGT |  |
| W909_15410-3 | ACCCCCACATTCTGGAGTAAGATCGATACTGCCAGCTAAC |  |
| W909_15410-4 | ggactagtAGTCTGAATACACCTGGCAG |  |
| W909_16285-1 | cgggatccTGCATTTGGGTGAGCGTTAC | *W909_16285* deletion |
| W909_16285-2 | TTTCAACCCCTTCGGCTACCAGGATGCCCTGAAACAGACT |  |
| W909_16285-3 | AGTCTGTTTCAGGGCATCCTGGTAGCCGAAGGGGTTGAAA |  |
| W909_16285-4 | ggactagtCGAAGAGAAAGCCTTTGTGC |  |
| W909_16555-1 | cgggatccAAAACGGAAGAAGCGTGTCG | *W909_16555* deletion |
| W909_16555-2 | ACAGTGCGTTATCTGCCGTTCCATTTCTGGTGGCACAAGA |  |
| W909_16555-3 | TCTTGTGCCACCAGAAATGGAACGGCAGATAACGCACTGT |  |
| W909_16555-4 | ggactagtACGGATTTGCGTCAGGTGAA |  |
| W909_17280-1 | cgggatccTCAGCACCAGATGACAACCA | *W909_17280* deletion |
| W909_17280-2 | CCGTTGTTGTTTCTGTCATGGTGTAGCCGTTATTCACGTG |  |
| W909_17280-3 | CACGTGAATAACGGCTACACCATGACAGAAACAACAACGG |  |
| W909_17280-4 | ggactagtCACGAAGACCCAGGTGTTAT |  |
| W909_18445-1 | cgggatccGGACAGGTCATCATCAGGAT | *W909_18445* deletion |
| W909_18445-2 | TAGAGCAGGTGGCTGTGTAAGTAGGCGATTCCGTGAAAGA |  |
| W909_18445-3 | TCTTTCACGGAATCGCCTACTTACACAGCCACCTGCTCTA |  |
| W909_18445-4 | ggactagtAATGGTCTGCGTAATCTGCG |  |
| W909_20210-1 | cgggatccGCTCAATAAAAGCAGGTCGG | *W909_20210* deletion |
| W909_20210-2 | CGTGGTGACCGTATTGATCACACTGCTTATCCTGGTCGAT |  |
| W909_20210-3 | ATCGACCAGGATAAGCAGTGTGATCAATACGGTCACCACG |  |
| W909_20210-4 | ggactagtCTGCTGTCTTACCATGTTGC |  |
| PKNG-F | GCCATCAAACCACGTCAAAT | pKNG101 plasmid  detection primer |
| PKNG-R | AACCAAGCCTATGCCTACAG |  |
| C10355-1 | cggaattcAGAGGGATAGTAGCCAGTTA | *W909_10355*  overexpression |
| C10355-2 | cgggatccCATCGCCTCATGAAGCATCA |  |
| C14945-1 | cccaagcttGCTCTTGGGGATTTCTACTG | *W909_14945*  overexpression |
| C14945-2 | cgggatccGCTAAAGCCAATACGACAGG |  |
| wspR-Hind III | cccaagcttCATGTACCACTCGCGTTCG | GGDEF domain  overexpression |
| wspR-EcoR I | cggaattcTCAGCCCGCCGGGGCC |  |
| rocR-Hind III | cccaagcttTGTGTGGACCGTGATG | EAL domain  overexpression |
| rocR-EcoR I | cggaattcTCAGGATCCGGAGCAATAGT |  |
| MCS-F | TCTTCGCTATTACGCCAGCT | pBBR1-MCS-4 plasmid  detection primer |
| MCS-R | GGCTCGTATGTTGTGTGGAA |  |
