# Supplementary material for: Genetic Modulation of c-di-GMP Turnover Affects Multiple Virulence Traits and Bacterial Virulence in Rice Pathogen Dickeya zeae
Source: PLoS One. 2016 Nov 17;11(11):e0165979. doi: 10.1371/journal.pone.0165979 (PMC5113947; doi:10.1371/journal.pone.0165979)
Supplement: S4 Table — (DOCX) [file pone.0165979.s007.docx]

**S4 Table. Homologous comparison of c-di-GMP metabolism proteins between *D. zeae* EC1 and *D. dadantii* 3937.**

| **Gene locus tag in EC1** | **Gene locus tag in**  ***D. dadantii* 3937** | **Homology of amino acids (%)** | **Product** |
| --- | --- | --- | --- |
| *W909_01375* | *Dda3937_00661* | 85% | CsrB/C sRNA degradation factor |
| *W909_02155* | *——* | —— | —— |
| *W909_06420* | *Dda3937_03276*(*gcpC*) | 81% | hypothetical protein |
| *W909_06670* | *Dda3937_00796* | 81% | GGDEF domain protein |
| *W909_07585* | *Dda3937_03022* | 79% | hypothetical protein |
| *W909_10355* | *Dda3937_03689*(*ecpB*) | 93% | Sensory box/GGDEF family protein |
| *W909_11190* | *Dda3937_00104*(*gcpD*) | 83% | Circadian input kinase A / Phytochrome-like protein |
| *W909_11910* | *Dda3937_03651* | 79% | hypothetical protein |
| *W909_11975* | *Dda3937_03639* | 86% | Rtn protein |
| *W909_14000* | *——* | —— | —— |
| *W909_14520* | *Dda3937_04330* | 76% | Response regulator |
| *W909_14945* | *Dda3937_03858*(*gcpA*) | 95% | hypothetical protein |
| *W909_14950* | *Dda3937_03856*(*ecpC*) | 88% | EAL domain containing protein involved in flagellar function |
| *W909_15410* | *Dda3937_01915* | 93% | Sensory box/GGDEF family protein |
| *W909_16285* | *——* | —— | —— |
| *W909_16555* | *Dda3937_03463* | 85% | GGDEF family protein |
| *W909_17280* | *Dda3937_00389* | 74% | GGDEF domain protein |
| *W909_18445* | *——* | —— | —— |
| *W909_20210* | *Dda3937_01101* | 88% | Sensory box protein |
